# FK506-Binding Protein like (FKBPL) Has an Important Role in Heart Failure with Preserved Ejection Fraction Pathogenesis with Potential Diagnostic Utility

**DOI:** 10.3390/biom13020395

**Published:** 2023-02-18

**Authors:** Michael Chhor, Hao Chen, Djurdja Jerotić, Milorad Tešić, Valentina N. Nikolić, Milan Pavlović, Rada M. Vučić, Benjamin Rayner, Chris J. Watson, Mark Ledwidge, Kenneth McDonald, Tracy Robson, Kristine C. McGrath, Lana McClements

**Affiliations:** 1 Faculty of Science, School of Life Sciences, University of Technology Sydney, Broadway, NSW 2007, Australia; 2 Faculty of Medicine, University of Belgrade, 11000 Belgrade, Serbia; 3 Clinic for Cardiology, University Clinical Center of Serbia, 11000 Belgrade, Serbia; 4 Department of Pharmacology and Toxicology, Faculty of Medicine, University of Nis, 18000 Nis, Serbia; 5 Department of Internal Medicine—Cardiology, Faculty of Medicine, University of Nis, 18000 Nis, Serbia; 6 Department of Internal Medicine, Faculty of Medical Sciences, University of Kragujevac, 34000 Kragujevac, Serbia; 7 Department of Cardiology, Clinical Centre of Kragujevac, 34000 Kragujevac, Serbia; 8 Inflammation Group, Heart Research Institute, University of Sydney, Sydney, NSW 2006, Australia; 9 Wellcome-Wolfson Institute for Experimental Medicine, Queen’s University Belfast, Belfast BT9 7BL, UK; 10 STOP-HF Unit, St. Vincent’s University Hospital, D04 T6F4 Dublin, Ireland; 11 School of Medicine, University College Dublin, D04 V1W8 Dublin, Ireland; 12 School of Pharmacy and Biomolecular Sciences, Royal College of Surgeons in Ireland, D02 YN77 Dublin, Ireland

**Keywords:** heart failure, biomarkers, heart failure with preserved ejection fraction, HFpEF, HCM, hypertrophic cardiomyopathy, FKBPL, plasma, angiotensin, AD-01

## Abstract

Heart failure (HF) is the leading cause of hospitalisations worldwide, with only 35% of patients surviving the first 5 years after diagnosis. The pathogenesis of HF with preserved ejection fraction (HFpEF) is still unclear, impeding the implementation of effective treatments. FK506-binding protein like (FKBPL) and its therapeutic peptide mimetic, AD-01, are critical mediators of angiogenesis and inflammation. Thus, in this study, we investigated—for the first time—FKBPL’s role in the pathogenesis and as a biomarker of HFpEF. In vitro models of cardiac hypertrophy following exposure to a hypertensive stimulus, angiotensin-II (Ang-II, 100 nM), and/or AD-01 (100 nM), for 24 and 48 h were employed as well as human plasma samples from people with different forms of HFpEF and controls. Whilst the FKBPL peptide mimetic, AD-01, induced cardiomyocyte hypertrophy in a similar manner to Ang-II (*p* < 0.0001), when AD-01 and Ang-II were combined together, this process was abrogated (*p* < 0.01–0.0001). This mechanism appears to involve a negative feedback loop related to FKBPL (*p* < 0.05). In human plasma samples, FKBPL concentration was increased in HFpEF compared to controls (*p* < 0.01); however, similar to NT-proBNP and Gal-3, it was unable to stratify between different forms of HFpEF: acute HFpEF, chronic HFpEF and hypertrophic cardiomyopathy (HCM). FKBPL may be explored for its biomarker and therapeutic target potential in HFpEF.

## 1. Introduction

Heart failure (HF) is a complex cardiovascular disease (CVD) that is characterised by a failure to meet circulatory demands [1]. Apart from genetic causes, common modifiable risk factors include obesity, diabetes mellitus, high blood pressure and smoking. Clinical symptoms include fatigue, weight gain, shortness of breath, and difficulty performing daily tasks [2]. Worldwide, HF is estimated to affect 40 million people annually [2]. In Australia, CVD is responsible for 25% of all mortalities, reaching an economic cost of 11.8 billion dollars per year [3].

HF diagnosis includes clinical symptoms, patient history and echocardiographic measurements [2]. Classification of HF into its phenotypes is based on the symptoms present and the left ventricular ejection fraction (EF). The European Society of Cardiology guidelines outline that an EF ≤ 40% is defined as heart failure with a reduced ejection fraction (HFrEF), an EF ≥ 50% as heart failure with a preserved ejection fraction (HFpEF) and an EF between 41–49% as heart failure with a mildly reduced ejection fraction (HFmrEF) [1,4]. Despite accounting for almost half the cases of HF, those with HFpEF have poorer management and prognosis compared to patients with HFrEF [5].

In conjunction with HF diagnosis, biomarker measurements provide crucial information surrounding the pathophysiology, severity and progression of HF [1]. Natriuretic peptides are the choice biomarkers to aid in such diagnosis—namely, brain natriuretic peptide (BNP) and N-terminal (NT)-pro hormone BNP (NT-proBNP), which are both reflective of myocardial stretch. Clinically, both BNP and NT-proBNP are reliable diagnostic and prognostic markers of HF. However, BNP levels have been shown to be elevated in cases of pulmonary and renal diseases, but are decreased in overweight patients [6]. NT-proBNP, in addition to having a longer half-life than BNP, has been shown to be less affected by parameters such as obesity—perhaps increasing its clinical utility [6]. Additionally, Galectin-3 is emerging as a promising biomarker of HFpEF [7]—the expression of which is positively correlated with adverse cardiac remodelling [8].

FK506-binding protein like (FKBPL) is a divergent member of the immunophilin family known for its role as a secreted anti-angiogenic protein that exhibits its action via CD44, establishing its critical role in angiogenesis [9,10]. Additionally, FKBPL has been shown to regulate steroid receptor and inflammatory signalling via CD44, HSP90 and STAT3, with an important regulatory function in vascular health [10,11,12]. AD-01 and ALM201 are FKBPL-based therapeutic peptides developed based on its anti-angiogenic domain, demonstrating effective anti-inflammatory and anti-angiogenic effects [13]. Even though full FKBPL knockout has been shown to be embryonically lethal, heterozygous knockdown of FKBPL in mice does not lead to any clinically detectable adverse phenotype; however, at the proteomic level, it shows early signs of endothelial dysfunction and impaired vascular integrity [10]. Recently, it was shown that FKBPL plasma concentrations are increased in the presence of CVD and the absence of diabetes mellitus compared to healthy controls, and FKBPL is positively correlated with the echocardiographic parameters of diastolic dysfunction [12]. However, its diagnostic or pathogenic role has not previously been demonstrated in HF. In light of these important functions associated with FKBPL, it is likely that it may have a role in the development of HF—particularly HFpEF—since inflammation and microvascular dysfunction are hallmark features of HFpEF [14]. Thus, this study evaluated the role of FKBPL in the development of cardiac hypertrophy and HFpEF using in vitro models of cardiomyoblasts exposed to a hypertensive stimulus, angiotensin-II (Ang-II), and/or the FKBPL mimetic AD-01, as well as human plasma samples from people with different forms of HFpEF and controls.

## 2. Methods and Materials

### 2.1. Cell Culture and Treatments

H9C2 rat cardiomyoblasts (Sigma Aldrich, Castle Hill, Australia) were cultured in Dulbecco’s Modified Eagle’s Medium (DMEM)(Thermofisher, Waltham, MA, USA), supplemented with 10% foetal bovine serum (FBS)(Thermofisher, Waltham, MA, USA). Cells were treated with Ang-II (100 nM)(Sigma Aldrich, Castle Hill, Australia), AD-01 (100 nM)(Sigma Aldrich, Castle Hill, Australia) or a combination of Ang-II and AD-01 for 48 h before measuring the cell/nucleus size and extracting RNA and protein.

### 2.2. Cell Size Analysis

The cell and nucleus size were determined using an Axio Imager A2 microscope (Carl Zeiss AG, Oberochen, Germany) and ZEISS Zen 2 imaging software (Carl Zeiss AG, Oberochen, German, v.1.0) at 20× magnification. ImageJ (National Institutes of Health, Bethesda, MD, USA) was used to measure and quantify cell/nucleus size.

### 2.3. Western Blot

Proteins were separated by molecular weight using sodium dodecyl sulfate polyacrylamide gel electrophoresis (SDS-PAGE). The loading buffer for the SDS-PAGE was Laemmli sample buffer (Bio-Rad Laboratories, Hercules, CA, USA) containing the reducing agent dithiothreitol (DTT), according to Laemmli (1970) [15]. The standard ladder used to estimate the molecular weight of the proteins was a Kaleidoscope protein ladder (Bio-Rad Laboratories, Hercules, CA, USA). FKBPL primary antibody (1:1000; in PBS; Proteintech, Rosemont, IL, USA) was used, alongside a ß-actin primary antibody (1:10,000; in PBS; Abcam, Cambridge, UK) to normalise the relative FKBPL concentration. The membrane was scanned using the ChemiDoc imaging system (Bio-Rad Laboratories, Hercules, CA, USA). The scanned pictures with peptide bands were processed through ImageJ for relative quantification.

### 2.4. Reverse Transcription-Polymerase Chain Reaction (RT-qPCR)

Total RNA was extracted from the treated cells using the ISOLATE II RNA Mini Kit (Bioline, Eveleigh, Australia), following the manufacturer’s guidelines. Reverse transcription was then performed using RT kit iScript Reverse transcription Supermix (Bio-Rad Laboratories, Hercules, CA, USA), before qPCR was performed using a SensiFAST SYBR No-ROX Kit (Bioline, Everleigh, Australia) and the primers listed for β-actin (FW: 5′-CGCGAGTACAACCTTCTTGC-3′ and RW: 5′-CGTCATCCATGGCGAACTGG-3′), FKBPL (FW: 5′-TGGCCTCTCAGGTCTGAACTA-3′ and RW: 5′-TGGGGACTGCTGCTTAATCG-3′), BNP (FW: 5′-TCCTTAATCTGTCGCCGCTG-3′ and RW: 5′-TCCAGCAGCTTCTGCATCG-3′) and ANP (FW: 5′-CTGGGACCCCTCCGATAGAT-3′ and RW: 5′-TTCGGTACCGGAAGCTGTTG-3′). Total mRNA expression levels were calculated using the 2^−ΔΔCT^ method, using β-actin as the reference gene.

### 2.5. Participants and Samples

A total of 33 patients diagnosed with HFpEF were enrolled in this study, according to the latest guidelines for HF [16]. Transthoracic echocardiography was performed and blood samples were collected from each participant at the time of the outpatient visit or hospital admission. Patients were excluded if there was a presence of significant valvular disease. Patients were divided into three sub-groups of HFpEF depending on their clinical symptoms: HCM (*n* = 15), acute HFpEF (*n* = 9) and chronic HFpEF (*n* = 9). A control group (*n* = 40) of participants who were high-risk for CVD, but without left ventricular diastolic dysfunction, were also included in this study (Table 1).

All participants provided written consent prior to inclusion and blood collection. This study was conducted in accordance with the Declaration of Helsinki and ethical approval was obtained from individual hospitals and institutions.

### 2.6. Plasma Marker Measurement

Blood samples collected from participants were centrifuged at 3000× *g* for 10 min to collect plasma. Plasma FKBPL concentrations were measured using an FKBPL ELISA assay (Cloud-Clone, Wuhan, China), following the manufacturer’s guidelines. Plasma NT-proBNP and Gal-3 concentrations were also measured using an ELISA (NT-proBNP, Abcam, Cambridge, UK; Gal-3, Elabscience, Wuhan, China). Gal-3 and NT-proBNP concentrations were not measured within the control group—comparisons were only performed between different HFpEF groups.

### 2.7. Statistical Analysis

All results are expressed as a mean ± SEM or SD. The data were checked for normal distribution before performing parametric tests (one-way ANOVA) with post-hoc multiple comparison testing. Correlations between two continuous variables were assessed based on the Pearson’s correlation coefficient. Statistical significance was defined as *p*  <  0.05 (two-sided). Statistical analyses were performed using SPSS software, version 24 (IBM Corp, Armonk, NY, USA) and GraphPad Prism v8.00 (Graphpad Software, Boston, MA, USA). Results with *p* < 0.05 were considered significant.

## 3. Results

### 3.1. FKBPL Peptide Mimetic, AD-01, and Angiotensin-II (Ang-II) Increase Cardiomyoblast Cell and Nucleus Size; However, AD-01 in the Presence of Ang-II Abrogates Ang-II-Induced Cardiac Hypertrophy

**Figure 1:**
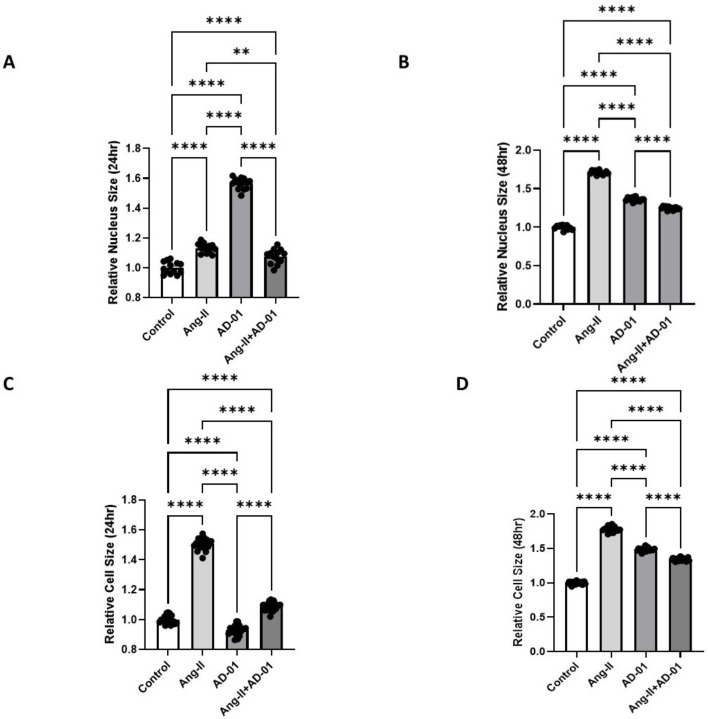
H9C2 cardiomyocyte cell size measurements following treatment with (i) Ang-II (100 nM), (ii) AD-01 (100 nM) and (iii) Ang-II (100 nM) + AD-01 (100 nM). (**A**) Relative nucleus size 24 h after treatments. (**B**) Relative nucleus size 48 h after treatments. (**C**) Relative cell size 24 h after treatments. (**D**) Relative cell size 48 h after treatments. Results expressed as Mean ± SEM (*n* = 6); One-way ANOVA with Tukey’s post-hoc; ** *p* < 0.01, **** *p* < 0.0001 against control; Ang-II—angiotensin II; AD-01—FKBPL-based therapeutic peptide.

Given that cardiac hypertrophy often leads to HFpEF, we determined the effect of a hypertensive stimuli, Ang-II, on the nucleus and cell size of cultured H9C2 cardiomyoblasts [17,18]. Cardiomyoblast nucleus and cell size were significantly increased following both 24 h and 48 h treatment with Ang-II compared to the control (Figure 1A–D, *p* < 0.0001). The effect on the nucleus size was more pronounced after the 48 h treatment with Ang-II (~70% increase) compared to the 24 h treatment (~13% increase). In the presence of AD-01 alone, nucleus size was also increased with both the 24 h (~60% increase) and 48 h treatment (~40% increase; Figure 1A,B, *p* < 0.0001). Interestingly, following the 24 h treatment with AD-01, cell size was modestly decreased (~7% decrease; Figure 1C, *p* < 0.0001), whereas the 48 h treatment with AD-01 led to an increase in cell size similar to that in the nucleus size (Figure 1D, *p* < 0.0001). When the AD-01 treatment was added to the Ang-II exposure, the increase in the nucleus size was abrogated both at 24 and 48 h (*p* < 0.01 and *p* < 0.0001, respectively; Figure 1A,B). The cardiomyoblast cell size was also abrogated when AD-01 was added to Ang-II both at 24 and 48 h (*p* < 0.0001); at both time points, AD-01 in the presence of Ang-II led to a ~30–40% reduction in cell size compared to Ang-II exposure alone (Figure 1C,D).

### 3.2. AD-01 Abrogates Ang-II-Induced Increases in FKBPL Protein Expression

**Figure 2:**
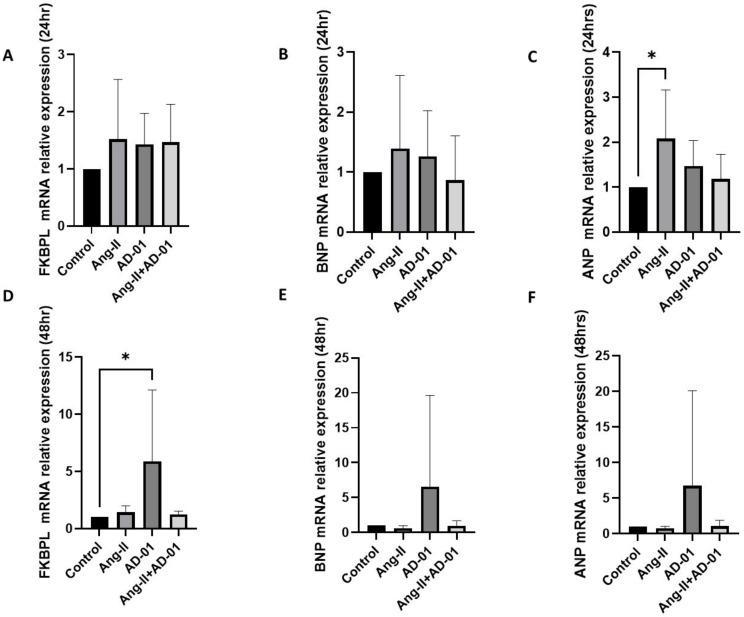
H9C2 cardiomyocyte mRNA expression of FKBPL, BNP and ANP following Ang-II and/or AD-01 treatment. H9C2 cells were exposed to treatment groups (i) Ang-II (100 nM), (ii) AD-01 (100 nM) and (iii) Ang-II (100 nM) + AD-01 (100 nM) for 24 or 48 h before RNA lysates were collected and qPCR performed. (**A**) FKBPL mRNA expression at 24 h; (**B**) BNP mRNA expression at 24 h; (**C**) ANP mRNA expression at 24 h; (**D**) FKBPL mRNA expression at 48 h; (**E**) BNP mRNA expression at 48 h; (**F**) ANP mRNA expression at 48 h. Results expressed as Mean ± SEM (*n* ≥ 4), One-way ANOVA with Tukey’s post-hoc. * *p* < 0.05. Ang-II—angiotensin II; AD-01—FKBPL-based therapeutic peptide.

Next, we determined FKBPL, BNP and ANP mRNA expression following 24 h treatment with Ang-II and/or AD-01. Apart from with ANP following Ang-II exposure, no significant change was obtained in the mRNA expression of any of the three genes (Figure 2A–C). Following 48 h exposure of H9C2 cells to Ang-II, AD-01 or Ang-II + AD-01, the only statistically significant change was observed in FKBPL mRNA expression after AD-01 treatment (*p* < 0.05), and although BNP and ANP mRNA expression showed a trend towards an increase, this was not statistically significant at 48 h (Figure 2D–F). The increase in all three genes (FKBPL, BNP and ANP) was the largest following 48 h treatment with AD-01, compared to Ang-II or Ang-II plus AD-01. AD-01 in the presence of Ang-II showed a much lower induction in gene expression than AD-01 alone although this was not statistically significant (Figure 2D–F).

**Figure 3:**
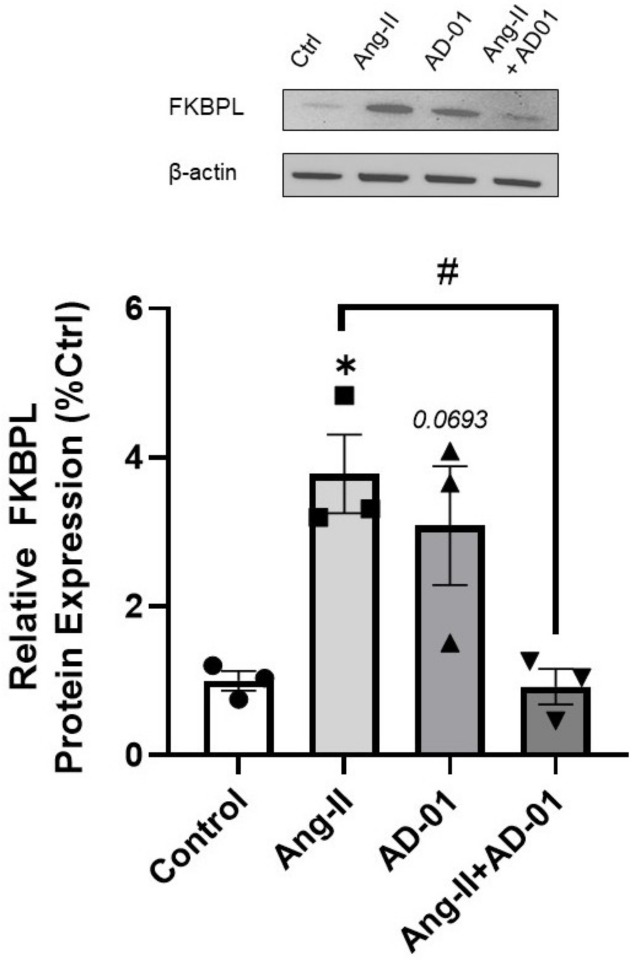
FKBPL protein expression in H9C2 cardiomyocytes following Ang-II and/or AD-01 treatment. H9C2 cells were exposed to treatment groups (i) Ang-II (100 nM), (ii) AD-01 (100 nM) and (iii) Ang-II (100 nM) + AD-01(100 nM) for 48 h. Relative FKBPL expression was measured. Results expressed as Mean ± SEM (*n* = 3); One-way ANOVA with Tukey’s post-hoc; * *p* < 0.05 against control; # *p* < 0.05 against Ang-II group. Ang-II—angiotensin II; AD-01—FKBPL-based therapeutic peptide.

Interestingly, at the protein level, cardiomyoblasts exposed to Ang-II for 48 h showed a significant increase in FKBPL expression compared to the control (Figure 3, *p* < 0.05), and although not significant, a trend towards increased FKBPL protein expressed was observed following AD-01 treatment (*p* = 0.07). In combination with Ang-II, AD-01 was able to abrogate Ang-II-induced FKBPL overexpression (Figure 3, *p* < 0.05).

### 3.3. FKBPL Plasma Concentration Is Increased in Patients with HFpEF but Does Not Differ between Subgroups

**Figure 4:**
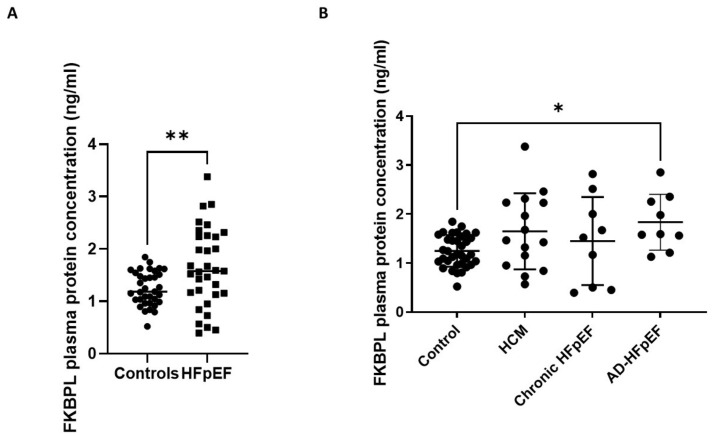
FKBPL plasma protein concentrations in patients with HFpEF. Patients were divided into subgroups based on HFpEF symptoms: HCM (*n* = 15), chronic HFpEF (*n* = 9) and acute decompensated HFpEF (*n* = 9). (**A**) FKBPL plasma concentration of combined HFpEF subgroups compared to controls (*n* = 40). (**B**) FKBPL plasma concentration within HFpEF subgroups, compared to controls. Results expressed as Mean ± SD; One-way ANOVA with Tukey’s post-hoc; * *p* < 0.05, ** *p* < 0.005. HCM—hypertrophic cardiomyopathy; HFpEF—chronic heart failure with preserved ejection fraction; AD-HFpEF—acute decompensated HFpEF.

The FKBPL plasma concentration was increased when all the HFpEF subgroups were combined together (1.645 ng/mL ± 0.75 SD) and compared to the controls (1.26 ng/mL ± 0.3 SD); Figure 4A, *p* < 0.01. However, when different HFpEF forms were separated into subgroups (acute, chronic and HCM), FKBPL plasma concentrations were only significantly increased in the acute HFpEF subgroup compared to the control (Figure 4B, *p* < 0.05), although there was a trend of increased FKBPL concentrations in HCM compared to controls (*p* = 0.07).

**Figure 5:**
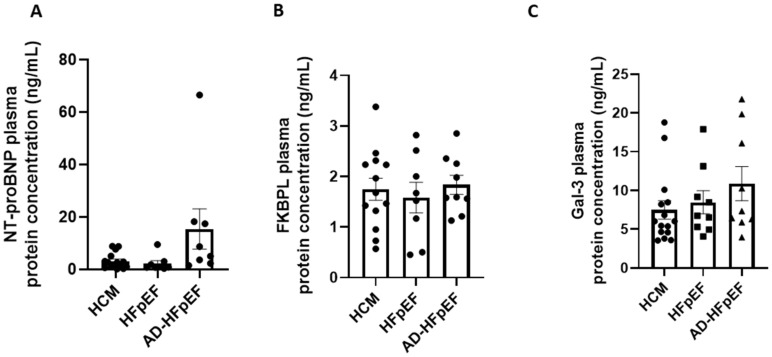
Biomarker plasma protein concentrations in subgroups of HFpEF. Patients were divided into subgroups based on HFpEF symptoms, HCM (*n* = 15), chronic HFpEF (*n* = 9) or acute decompensated HFpEF (*n* = 9). (**A**) NT-proBNP plasma concentration of HFpEF subgroups measured by ELISA. (**B**) FKBPL plasma concentration of HFpEF subgroups measured by ELISA. (**C**) Gal-3 plasma concentration of HFpEF subgroups measured by ELISA. Results expressed as Mean ± SEM, One-way ANOVA with Tukey’s post-hoc. HCM—hypertrophic cardiomyopathy; HFpEF—chronic heart failure with preserved ejection fraction; AD-HFpEF—acute decompensated HFpEF.

When FKBPL plasma concentrations were compared between different HFpEF forms, no significant differences were observed between HCM, acute and chronic HFpEF (Figure 5A). Interestingly, a well-established biomarker, NT-proBNP, and an emerging biomarker, Gal-3, also did not show significant differences between the three forms of HFpEF. Nevertheless, NT-proBNP showed a trend towards an increase in acute HFpEF compared to HCM (*p* = 0.08) or chronic HFpEF (*p* = 0.1).

### 3.4. FKBPL Is Positively Correlated with IVST, Indicative of Microvascular Dysfunction

Echocardiographic measurements are clinically used alongside symptomatic assessments of HF patients and biomarkers, providing key information on cardiac structure and function [4]. In this study we measured limited echocardiographic parameters including end-diastolic diameter (EDD), end-systolic diameter (ESD), posterior wall thickness (PWT) and intraventricular septal thickness (IVST); this is because we have previously shown correlations between FKBPL and echocardiographic parameters [12], whereas the aim of the study was to investigate FKBPL mechanisms in HFpEF patients specifically, in light of its significant role in vasculature function. Correlation analyses (Table 2) showed that FKBPL was positively correlated with IVST (r_s_ = 0.621, *p* < 0.000) and negatively correlated with ESD and PWT (r_s_ = −0.361, *p* = 0.042; r_s_ = −0.401 *p* = 0.021). There was no significant correlation between FKBPL and NT-proBNP or Gal-3 (Table 3). NT-proBNP and Gal-3 showed a positive correlation between each other (r_s_ = 0.464, *p <* 0.007).

## 4. Discussion

HF pathophysiology is complex and involves various mechanistic pathways as part of its development and progression. Changes in cardiomyocyte cell morphology and function play a key role in the progression of the key mechanisms and processes involved in HF pathogenesis [19]. The renin-angiotensin-aldosterone system (RAAS) is activated by hypovolemia and the sympathetic nervous system. The main product of the RAAS is Ang-II, which has compensatory systemic effects that, if they persist, can exacerbate HF. This is because, in HF, Ang-II is stimulated to maintain cardiac output through increased vasoconstriction, salt retention, contractility, and the activation of inflammatory mediators [1,20,21]. The neuroendocrine pathological mechanisms of HF are regulated by the sympathetic nervous system and are linked to the RAAS [21]. Ang-II has been implicated in adverse cardiac remodelling and leads to an increase in interstitial fibrosis, contributing to HF [1]. Adverse cardiac remodelling through hypertrophy, besides physical alterations, modulates gene expression and the viability of cardiomyocytes, which may contribute to cardiac dysfunction and HF [19]. Interestingly, a recent report demonstrated that the presence of adverse cardiac remodelling in HFpEF patients is associated with worse outcomes compared to those without adverse remodelling [22].

Our findings in this study reveal an interesting mechanism involving Ang-II and FKBPL-based peptide therapeutic, AD-01, when examining their effects on cell and nucleus size. Ang-II or AD-01 treatment led to a significant increase in both cell and nucleus size at 24 and 48 h, with Ang-II and AD-01 displaying similar trends—except in terms of cell size following 24 h treatment. Interestingly, when these two treatments were combined, Ang-II and AD-01 exhibited a significant decrease in cell and nucleus size compared to individual treatments, akin to the size of the control group. Consistent with these findings, 48 h treatment with Ang-II or AD-01 increased the protein expression of FKBPL, which was again abolished when combining these two treatments together. FKBPL plays a critical role in developmental and pathological angiogenesis and vascular function, which has been demonstrated in previous studies in which a murine homozygous knockout of FKBPL was embryonically lethal, whereas heterozygous knockdown resulted in impaired vascular integrity [10,11,23]. Furthermore, FKBPL has been shown to operate via the STAT3 [13], CD44 [24] and nuclear factor kappa B (NF-kB) [9] inflammatory pathways that commonly underly HF pathophysiology [25]. Thus, vascular dysfunction due to aberrant endothelial cell homeostasis, pro-inflammatory signalling and restricted angiogenesis potentially implicate FKBPL in the development of HF. Our findings suggest that AD-01 may exacerbate hypertrophy within cardiomyocytes—likely via FKBPL. However, there exists a compensatory mechanism when Ang-II is present; AD-01 abrogates this effect via a negative feedback mechanism to reverse the hypertrophic effect. As an FKBPL mimetic, AD-01 has been shown previously and, in this study, to increase FKBPL mRNA and protein expression when used alone [24]; this mechanism is altered in the presence of Ang-II, whereby FKBPL expression is normalised. These findings present a complex and compensatory mechanism of AD-01 as a FKBPL mimetic, in producing an anti-hypertrophic effect in Ang-II-induced myopathy that needs to be further studied.

In evaluating the biomarker potential of FKBPL in HFpEF, NT-proBNP and Gal-3 plasma concentrations were also measured in this study. NT-proBNP has been well-established in the clinical diagnosis of HF [4], whereas Gal-3—although not clinically used—has been presented in recent literature as a promising biomarker candidate for the diagnosis of HFpEF [7,26]. Gal-3′s diverse functionality in inflammation contributes to myocardial remodelling and fibrosis [8], where the inhibition of Gal-3 has been reported to ameliorate these conditions [27]. Previous reports have shown that FKBPL plasma concentrations are increased in the presence of CVD [12] and in the absence of diabetes mellitus, compared to healthy controls. FKBPL is also positively correlated with parameters of diastolic dysfunction including left atrium volume and size, IVST at the end of diastole and deceleration time [12]. In the same study, FKBPL was positively correlated with a clinically used marker of HFpEF, BNP, and it was one of the determinants of CVD in conjunction with age, gender, total-cholesterol, and systolic blood pressure (SBP) [12]. Here, we showed that the FKBPL plasma concentration was significantly increased between the control group and patients with HFpEF, implicating FKBPL’s possible role as a biomarker for HFpEF. In further evaluating the biomarker potential of FKBPL in HFpEF, FKBPL plasma concentrations were found to be significantly increased when comparing the control group to acute HFpEF and only showed an increasing trend in HCM—suggesting a mechanistic role for FKBPL in the pathophysiology and progression of HFpEF. Previous studies have shown that in a murine model of HFpEF, deletion of STAT3 in cardiomyocytes resulted in the manifestation of the clinical characteristics of HFpEF [28]. Given that FKBPL is increased in HFpEF patients, and that it inhibits the inflammatory STAT3 pathway [13], this mechanism may contribute towards HFpEF pathophysiology.

When comparing different forms of HFpEF, our study found no significant differences in the plasma concentrations of FKBPL, NT-proBNP or Gal-3. Therefore, none of the examined biomarkers have shown to be able to stratify between specific forms of HFpEF in this study. FKBPL has previously been reported to be positively correlated with BNP [11]; however, we found no correlation with either NT-proBNP or Gal-3, whereas the latter two were positively correlated with each other. This is likely due to the diverse role of FKBPL in HFpEF, which is independent of NT-proBNP and Gal-3, and it might contribute to different pathogenic processes and mechanisms involved in microvascular dysfunction, inflammation and restricted angiogenesis. This could also be specific to our patient samples.

In patients with HCM, the presence of microvascular dysfunction has been recognized as a strong predictor of clinical deterioration and mortality [29,30]. In fact, myocardial wall thickness is the strongest predictor of reduced global hyperaemic myocardial blood flow in HCM [31]. Subsequently, there is a higher probability of the development of myocardial fibrosis in segments with reduced hyperaemic myocardial blood flow [32]. Our study demonstrated a clinically relevant positive correlation between FKBPL and IVST, likely implicating FKBPL in the microvascular dysfunction of the LV hypertrophy, which is related to the pathogenesis of HFpEF [33,34]. This was also confirmed in the in vitro part of the study where the FKBPL peptide mimetic, AD-01, induced cardiomyoblast hypertrophy whilst also increasing FKBPL expression.

The limitations of this study include the cross-sectional nature of the study in terms of the recruited controls and modest patient numbers. Nevertheless, we included well-known biomarkers of HFpEF—NT-proBNP and Gal-3—as a comparison and supported the findings with in vitro models of HFpEF that aligned with the clinical sample findings, showing that FKBPL is positively correlated with HFpEF and, potentially, its progression.

## 5. Conclusions

In this study, we demonstrated for the first time that FKBPL may be implicated in HFpEF. An FKBPL-based peptide therapeutic, AD-01, was able to abrogate Ang-II-induced FKBPL upregulation and cardiomyoblasts hypertrophy. Aligned to this, FKBPL human plasma levels were increased in HFpEF compared to controls; however, FKBPL was unable to distinguish between different forms of HFpEF, similar to NT-proBNP and Gal-3. Finally, FKBPL was positively correlated with an echocardiography parameter reflective of cardiac microvascular dysfunction and hypertrophy, further strengthening the evidence for its role in the pathogenesis of HFpEF.

## Figures and Tables

**Table 1:** Patient groups and clinical characteristics.

Characteristics	Controls (*n* = 40)	Acute HFpEF (*n* = 9)	Chronic HFpEF (*n* = 9)	HCM (*n* = 15)
Age (years)	72.43 ± 6.4	73.4 ± 13.3	64.6 ± 10.6	50.7 ± 13.6
Female (no. [%])	13 (37.1)	4 (44.4)	3 (33.3)	3 (20)
BMI (kg/m^2^)	27.6 ± 5.3	32 ± 4.4	28 ± 2.5	25.9 ± 4.1
EF (%)	n/a	57.6 ± 10.9	57.4 ± 8.0	64.5 ± 3.8
NYHA Class	n/a	I/II/III	I/II	I/II
Diabetes n (%)	20 (54)	5 (56)	2 (22)	0 (0)
NT-proBNP (ng/mL)	n/a	13.8 ± 20.9	2.3 ± 3.0	3.2 ± 3.0
FKBPL (ng/mL)	1.26 ± 0.3	1.8 ± 0.6	1.5 ± 0.9	1.6 ± 0.8
Gal-3 (ng/mL)	n/a	10.9 ± 6.6	8.5 ± 4.5	7.5 ± 4.6
	Echocardiography measurement			
EDD (mm)	n/a	55.0 ± 11.6	52.8 ± 6.9	47.5 ± 5.5
ESD (mm)	n/a	37 ± 9.6	35.3 ± 8.2	28.9 ± 4.2
IVST (mm)	n/a	12.3 ± 2.9	12.4 ± 2.4	17.9 ± 2.3
PWT (mm)	n/a	11.7 ± 2.1	12.1 ± 1.5	9.3 ± 1.7
	Medications			
Aspirin (no. [%])	n/a	7 (78)	4 (44)	1 (7)
Purinergic receptor antagonists (no. [%])	n/a	5 (56)	3 (33)	0
Statins (no. [%])	n/a	6 (67)	3 (33)	2 (13)
Isosorbide mononitrate (no. [%])	n/a	3 (33)	1 (11)	0
Beta-blockers (no. [%])	n/a	9 (100)	6 (67)	14 (93)
ACE-inhibitors (no. [%])	n/a	7 (78)	5 (56)	4 (27)
Diuretics (no. [%])	n/a	4 (44)	4 (44)	4 (27)
Calcium channel blockers (no. [%])	n/a	3 (33)	2 (22)	1 (7)
Warfarin (no. [%])	n/a	1 (11)	1 (11)	0
Amiodarone (no. [%])	n/a	0	0	1 (7)
PPIs (no. [%])	n/a	4 (44)	3 (33)	0
Trimetazidine (no. [%])	n/a	1 (11)	1 (11)	0
Molsidomine (no. [%])	n/a	1 (11)	1 (11)	0
Spironolactone (no. [%])	n/a	0	3 (33)	0
Allopurinol (no. [%])	n/a	0	1 (11)	0
Aminophylline (no. [%])	n/a	0	2 (22)	0

n/a—not applicable; BMI, body mass index; HF, heart failure; HFpEF, heart failure with preserved ejection fraction; EDD, end-diastolic dimension; EF, ejection fraction; ESD, end-systolic dimension; IVST, intraventricular septal thickness; PWT, posterior wall thickness; NT-proBNP, N-terminal pro-B-type natriuretic peptide; and NYHA, New York Heart Association Functional Classification; PPIs, proton pump inhibitors.

**Table 2:** Correlations between FKBPL and echocardiography parameters.

		FKBPL	EDD	ESD	IVST	PWT
FKBPL	Pearson Correlation	1	−0.281	−0.361 *	0.621 ***	−0.401 *
	Sig. (2-tailed)		0.119	**0.042**	**0.000**	**0.021**
	N	33	32	32	33	33

Two-tailed test, * *p* < 0.05, *** *p* < 0.001.

**Table 3:** Pearson’s correlations between FKBPL, NT-proBNP and Gal-3.

		FKBPL	NT-proBNP	Gal-3
FKBPL	Pearson Correlation	1	0.063	−0.042
	Sig. (2-tailed)		0.731	0.815
	N	33	32	33
NT-proBNP	Pearson Correlation	0.063	1	0.464 **
	Sig. (2-tailed)	0.731		**0.007**
	N	32	32	32
Gal-3	Pearson Correlation	−0.042	0.464 **	1
	Sig. (2-tailed)	0.815	**0.007**	
	N	33	32	33

Two-tailed test, ** *p* < 0.01.

## Data Availability

The data presented in this study are available on request from the corresponding author.

## References

[1] Malik A., Brito D., Vaqar S., Chhabra L. (2022). Congestive Heart Failure.

[2] Baman J.R., Ahmad F.S. (2020). Heart Failure. JAMA.

[3] Australian Institute of Health and Welfare Heart, Stroke and Vascular Disease: Australian Facts.

[4] McDonagh T.A., Metra M., Adamo M., Gardner R.S., Baumbach A., Böhm M., Burri H., Butler J., Čelutkienė J., Chioncel O. (2021). 2021 ESC Guidelines for the diagnosis and treatment of acute and chronic heart failure. Developed by the Task Force for the diagno-sis and treatment of acute and chronic heart failure of the European Society of Cardiology (ESC) With the special contribution of the Heart Failure Association (HFA) of the ESC. Eur. Heart J..

[5] Inamdar A.A., Inamdar A.C. (2016). Heart Failure: Diagnosis, Management and Utilization. J. Clin. Med..

[6] Lewis R.A., Durrington C., Condliffe R., Kiely D.G. (2020). BNP/NT-proBNP in pulmonary arterial hypertension: Time for point-of-care testing?. Eur. Respir. Rev..

[7] Chen H., Chhor M., Rayner B.S., McGrath K., McClements L. (2021). Evaluation of the diagnostic accuracy of current biomarkers in heart failure with preserved ejection fraction: A systematic review and meta-analysis. Arch. Cardiovasc. Dis..

[8] Andrejic O.M., Vucic R.M., Pavlovic M., McClements L., Stokanovic D., Jevtovic–Stoimenov T., Nikolic V.N. (2019). Association between Galectin-3 levels within central and peripheral venous blood, and adverse left ventricular remodelling after first acute myocardial infarction. Sci. Rep..

[9] Annett S., Spence S., Garciarena C., Campbell C., Dennehy M., Drakeford C., Lai J., Dowling J., Moore G., Yakkundi A. (2021). The immunophilin protein FKBPL and its peptide derivatives are novel regulators of vascular integrity and inflammation via NF-κB signaling. bioRxiv.

[10] Yakkundi A., Bennett R., Hernández-Negrete I., Delalande J.-M., Hanna M., Lyubomska O., Arthur K., Short A., McKeen H., Nelson L. (2015). FKBPL Is a Critical Antiangiogenic Regulator of Developmental and Pathological Angiogenesis. Arter. Thromb. Vasc. Biol..

[11] Alqudah A., Eastwood K.-A., Jerotic D., Todd N., Hoch D., McNally R., Obradovic D., Dugalic S., Hunter A.J., Holmes V.A. (2021). FKBPL and SIRT-1 Are Downregulated by Diabetes in Pregnancy Impacting on Angiogenesis and Endothelial Function. Front. Endocrinol..

[12] Januszewski A.S., Watson C.J., O’Neill V., McDonald K., Ledwidge M., Robson T., Jenkins A.J., Keech A.C., McClements L. (2020). FKBPL is associated with metabolic parameters and is a novel determinant of cardiovascular disease. Sci. Rep..

[13] Annett S., Moore G., Short A., Marshall A., McCrudden C., Yakkundi A., Das S., McCluggage W.G., Nelson L., Harley I. (2019). FKBPL-based peptide, ALM201, targets angiogenesis and cancer stem cells in ovarian cancer. Br. J. Cancer.

[14] Chen H., Tesic M., Nikolic V.N., Pavlovic M., Vucic R.M., Spasic A., Jovanovic H., Jovanovic I., Town S.E.L., Padula M.P. (2022). Systemic Biomarkers and Unique Pathways in Different Phenotypes of Heart Failure with Preserved Ejection Fraction. Biomolecules.

[15] Laemmli U.K. (1970). Cleavage of Structural Proteins during the Assembly of the Head of Bacteriophage T4. Nature.

[16] Ponikowski P., Voors A., Anker S., Bueno H., Cleland J., Coats A., Falk V., González-Juanatey J.R., Harjola V.-P., Jankowska E. (2016). 2016 ESC Guidelines for the diagnosis and treatment of acute and chronic heart failure: The Task Force for the diagnosis and treatment of acute and chronic heart failure of the European Society of Cardiology (ESC)Developed with the special contribution of the Heart Failure Association (HFA) of the ESC. Eur. Heart J..

[17] Van Heerebeek L., Hamdani N., Handoko M.L., Falcao-Pires I., Musters R.J., Kupreishvili K., Ijsselmuiden A.J., Schalkwijk C.G., Bronzwaer J.G., Diamant M. (2008). Diastolic stiffness of the failing diabetic heart: Importance of fibrosis, advanced glycation end products, and myocyte resting tension. Circulation.

[18] Watkins S.J., Borthwick G.M., Arthur H.M. (2011). The H9C2 cell line and primary neonatal cardiomyocyte cells show similar hypertrophic responses in vitro. Vitr. Cell. Dev. Biol. Anim..

[19] Peter A.K., Bjerke M.A., Leinwand L.A. (2016). Biology of the cardiac myocyte in heart disease. Mol. Biol. Cell.

[20] Nakano S., Muramatsu T., Nishimura S., Senbonmatsu T. (2012). Cardiomyocyte and Heart Failure. Current Basic and Pathological Approaches to the Function of Muscle Cells and Tissues—From Molecules to Humans.

[21] Orsborne C., Chaggar P.S., Shaw S.M., Williams S.G. (2016). The renin-angiotensin-aldosterone system in heart failure for the non-specialist: The past, the present and the future. Postgrad. Med. J..

[22] Xu L., Pagano J., Chow K., Oudit G.Y., Haykowsky M.J., Mikami Y., Howarth A.G., White J.A., Howlett J.G., Dyck J.R. (2021). Cardiac remodelling predicts outcome in patients with chronic heart failure. ESC Heart Fail..

[23] Todd N., McNally R., Qudhah A., Jerotic D., Suvakov S., Obradovic D., Hoch D., Hombrebueno J.R., Campos G.L., Watson C.J. (2020). Role of A Novel Angiogenesis FKBPL-CD44 Pathway in Preeclampsia Risk Stratification and Mesenchymal Stem Cell Treatment. J. Clin. Endocrinol. Metab..

[24] Yakkundi A., McCallum L., O’Kane A., Dyer H., Worthington J., McKeen H.D., McClements L., Elliott C., McCarthy H., Hirst D.G. (2013). The Anti-Migratory Effects of FKBPL and Its Peptide Derivative, AD-01: Regulation of CD44 and the Cytoskeletal Pathway. PLoS ONE.

[25] Zeng H., Chen J.-X. (2019). Microvascular Rarefaction and Heart Failure With Preserved Ejection Fraction. Front. Cardiovasc. Med..

[26] De Boer R.A., Edelmann F., Cohen-Solal A., Mamas M.A., Maisel A., Pieske B. (2013). Galectin-3 in heart failure with preserved ejection fraction. Eur. J. Heart Fail..

[27] Zhong X., Qian X., Chen G., Song X. (2018). The role of galectin-3 in heart failure and cardiovascular disease. Clin. Exp. Pharmacol. Physiol..

[28] Zhao W., Chen Y., Yang W., Han Y., Wang Z., Huang F., Qiu Z., Yang K., Jin W. (2020). Effects of Cardiomyocyte-Specific Deletion of STAT3–A Murine Model of Heart Failure With Preserved Ejection Fraction. Front. Cardiovasc. Med..

[29] Cecchi F., Olivotto I., Gistri R., Lorenzoni R., Chiriatti G., Camici P.G. (2003). Coronary Microvascular Dysfunction and Prognosis in Hypertrophic Cardiomyopathy. N. Engl. J. Med..

[30] Tesic M., Beleslin B., Giga V., Jovanovic I., Marinkovic J., Trifunovic D., Petrovic O., Dobric M., Aleksandric S., Juricic S. (2021). Prognostic Value of Transthoracic Doppler Echocardiography Coronary Flow Velocity Reserve in Patients With Asymmetric Hypertrophic Cardiomyopathy. J. Am. Heart Assoc..

[31] Bravo P.E., Pinheiro A., Higuchi T., Rischpler C., Merrill J., Santaularia-Tomas M., Abraham M.R., Wahl R.L., Abraham T.P., Bengel F.M. (2012). PET/CT Assessment of Symptomatic Individuals with Obstructive and Nonobstructive Hypertrophic Cardiomyopathy. J. Nucl. Med..

[32] Ismail T.F., Hsu L.-Y., Greve A.M., Gonçalves C., Jabbour A., Gulati A., Hewins B., Mistry N., Wage R., Roughton M. (2014). Coronary microvascular ischemia in hypertrophic cardiomyopathy—A pixel-wise quantitative cardiovascular magnetic resonance perfusion study. J. Cardiovasc. Magn. Reson..

[33] Yang Y., Li Z., Guo X., Zhou Y., Chang Y., Yang H., Yu S., Ouyang N., Chen S., Sun G. (2022). Interventricular Septum Thickness for the Prediction of Coronary Heart Disease and Myocardial Infarction in Hypertension Population: A Prospective Study. J. Clin. Med..

[34] Kansal S., Roitman D., Sheffield L.T. (1979). Interventricular septal thickness and left ventricular hypertrophy. An echocardiographic study. Circulation.

[35] Ledwidge M., Gallagher J., Conlon C., Tallon E., O’Connell E., Dawkins I., Watson C., O’Hanlon R., Bermingham M., Patle A. (2013). Natriuretic peptide-based screening and collaborative care for heart failure: The STOP-HF randomized trial. JAMA.

[36] Tonry C., McDonald K., Ledwidge M., Hernandez B., Glezeva N., Rooney C., Morrissey B., Pennington S.R., Baugh J.A., Watson C.J. (2021). Multiplexed measurement of candidate blood protein biomarkers of heart failure. ESC Heart Fail..

[37] Watson C.J., Gallagher J., Wilkinson M., Russell-Hallinan A., Tea I., James S., O’Reilly J., O’Connell E., Zhou S., Ledwidge M. (2021). Biomarker profiling for risk of future heart failure (HFpEF) development. J. Transl. Med..

